# Long-term persistent infection of HPV 16 E6 up-regulate SP1 and hTERT by inhibiting LKB1 in lung cancer cells

**DOI:** 10.1371/journal.pone.0182775

**Published:** 2017-08-16

**Authors:** Jing-Hua Yang, Xiao-Yan Li, Xin Wang, Wei-Jian Hou, Xue-Shan Qiu, En-Hua Wang, Guang-Ping Wu

**Affiliations:** 1 Department of Pathology, The First Affiliated Hospital and College of Basic Medical Sciences, China Medical University, Shenyang, China; 2 Department of Pathology, Cancer Hospital of China Medical University, Shenyang, China; 3 Department of Tissue Engineering, College of Basic Medical Sciences, China Medical University, Shenyang, China; University of South Alabama Mitchell Cancer Institute, UNITED STATES

## Abstract

HPV 16 E6 upregulates hTERT expression in lung cancer cells. However, the underlying molecular mechanism is unclear. In this paper, E6, LKB1, SP1, and hTERT mRNA expression levels were detected in brushing cells of patients with lung cancer (n = 106) and with benign lung disease (n = 68) by qRT-PCR. The mRNA expression levels of E6, SP1, and hTERT were significantly increased in the malignant group compared with the benign group (*P* < 0.01). Conversely, the mRNA expression level of LKB1 was significantly decreased in the malignant group (*P* < 0.01). Furthermore, the correlation between E6, Sp1, hTERT, and LKB1 was performed, our results indicated that E6, Sp1, and hTERT with positive, but LKB1 with negative correlation (*P* < 0.01). To investigate the potential relationship between these genes, using double directional genetic manipulation, we showed that overexpression of E6 in H1299 cells down-regulated LKB1 mRNA and protein expression but up-regulated SP1 and hTERT as well as the transcriptional activity of Sp1. In contrast, knockdown of E6 in A549 cells by short-interference RNAs (siRNAs) up-regulated LKB1 expression, but down-regulated SP1 and hTERT expression as well as Sp1 activity. LKB1 loss upregulated both SP1 and hTERT at the protein and mRNA level as well as SP1 activity. To verify that the role of E6 on hTERT was mediated by SP1, siRNA knockdown of SP1 was performed on both H1299 and A549 cell lines. Inhibition of SP1 downregulated hTERT expression. Our results indicate that HPV16 E6 indirectly upregulated the expression of hTERT by inhibition of LBK1 expression and upregulation of Sp1 expression, thus suggesting a HPV-LKB1-SP1-hTERT axis for the tumorigenesis of lung cancer. Our study also provides new evidence to support the critical role of SP1 and LKB1 in the pathogenesis of HPV-related lung cancer, and suggests novel therapeutic targets.

## Introduction

Human papillomaviruses (HPV) is a cyclic double stranded DNA virus, which is associated with a variety of human diseases. The persistent infection of high-risk HPV is closely related, for instance, to the occurrence of cervical cancer [1]. In 1979, Syrjanen first hypothesized that HPV infection might play an important role in the occurrence of lung cancer [2]. Since then, a great deal of epidemiological evidence has confirmed this hypothesis [3–9]. With the rapid development of molecular biology, it has been found that high risk type HPV16 is the most common type of infection in the development of lung cancer [3,6], and that E6 and E7 proteins in HPV16 are the main oncogenes in the development of lung cancer [10], These studies support the notion that high risk HPV16 infection plays an important role in the development of non small cell lung cancer. However, HPV infection alone is not sufficient to cause tumor occurrence, because most patients with HPV infection have a natural prognosis after transient epithelial abnormalities. Only after long-term persistent infection is possible to cause lung cancer. As to the specific reasons and the possible mechanism, it is not clear at present. Recently, it was discovered that high risk HPV16 infection was closely related to the inactivation of tumor suppressor gene LKB1 in the occurrence of cervical cancer [11], and other researchers found that the LKB1 inactivation was closely related to the occurrence of lung cancer [12, 13]. We hypothesized that high risk HPV16 infection may also be associated with inactivation of LKB1 in the development of lung cancer. Therefore, it is necessary to link HPV16 and LKB1 together to explore the combined role and the molecular mechanism in the occurrence of lung cancer, to search for genes and their regulation of promoting cancer progression, and to provide a new approach for the treatment of HPV related lung cancer.

Liver kinase B1(LKB1), also known as serine/threonine kinase 11(STK11), is the tumor suppressor gene found for the first time in patients with Peutz-Jeghers syndrome (PJS) [14, 15]. LKB1 has serine / threonine protein kinase activity, regulates gene expression by phosphorylation of substrate proteins or binding to target proteins, and plays an important role in the development of lung cancer [16, 17]. Liang et al reported that by transfected LKB1 to LKB1-null human lung cell line A594, the overexpression of the LKB1 protein was strongly associated with a decrease in both expression and activity of transcription factor specificity protein 1 (SP1) [18].

SP1, a member of the SP proteins family, constitutes a group of highly conserved transcription factors present in a wide range of organisms. Their structures are defined by the presence of three highly conserved DNA-binding zinc finger domains which bind to similar, yet distinct, GC-rich target sequences. Recently, we found overexpression of SP1 mRNA in the bronchial brushing cells of patients with lung cancer [19]. Hedrick et al demonstrated that individual knockdown of SP1 by RNAi in lung cancer cell lines A549 resulted in inhibition of cell growth, decreased survival, and inhibition of migration/invasion [20]. Triptolide also inhibits transcription of hTERT through down-regulation of transcription factor specificity protein 1 in primary effusion lymphoma cells [21]. However, it is not clear whether E6 upregulates hTERT expression in lung cancer cells by HPV-LKB1-SP1-hTERT axis.

The main purpose of the study aims to evaluate diagnostic utility and correlation among E6, LKB1, SP1, and hTERT as tumor markers in brushing cells of patients with lung cancer. In particular, we aimed to evaluate the underlying molecular mechanism by which overexpression of E6 upregulates hTERT expression and might play an important role in the development of lung cancer.

## Materials and methods

The study was conducted according to the guidelines of the institutional review boards at the First Affiliated Hospital of China Medical University, we have obtained internal review board approval and/or patients informed consent for this study. A total of 106 brushing cells of patients with lung cancer who attended the laboratory of cytopathology at the First Affiliated Hospital of China Medical University during the period 2013–2014 were included in the study. There were 88 males (83.0%) and 18 females (17.0%), with a mean age of 64.3 years (range 40–79). Of the malignant cells, there were 20 adenocarcinomas (AC) and 86 squamous cell carcinomas (SCC). These 106 patients, as well as 68 randomly selected patients without lung cancer (58 with inflammation and 10 with endobronchial tuberculosis), were included as study subjects and control subjects, respectively. These all had biopsies, resections or clinical follow-up negative for malignancy.

All bronchoscopies were performed by two experienced bronchoscopists. Forceps biopsies and brushings were obtained from all subjects, including three to four forceps biopsies and two endobronchial brushings. In order to obtain sufficient numbers of cells in the brushings, these were taken before the forceps biopsies. Endobronchial brushings were transferred to a small vial containing SurePath^™^ preservative fluid (BD Tripath, Burlington NC, USA). A mucolytic agent (1 mL, BD Tripath) was added to the brushings in SurePath^™^ preservative fluid, incubated at room temperature for 30 minutes and vortexed for 10 seconds. Additional mucolytic agent was added to the mixture until the mucus was completely lysed. The mixture was transferred to a 10 mL tube and centrifuged at 2000 g for 10 minutes. The supernatant was removed and the pellet was resuspended in 7.5 mL of distilled water. This suspension was vortexed again and centrifuged at 2000 g for 5 minutes. The supernatant was removed and the pellet was vortexed and transferred to a AutoCyte PREP system (BD Tripath) for automatic preparation and staining of slides. Cytology was recorded as positive if malignant cells were reported. Specimens that were reported as ‘atypical’ have been included as ‘negative’ in the present study, because the patients with uncertain results cannot be treated as malignant in clinical practice. The residual material was used for RNA extraction.

### Cell culture

Based on our previous screening results for lung cancer cell lines, H1299 was selected as the representative of E6-low cell lines, whereas A549 was selected as the representative of E6-high cell lines, respectively. They were selected in the following transfection and interference assays. A549 and H1299 cell lines were obtained from the ATCC (Manassas, VA, USA). A549 cells were grown in Dulbecco's modified Eagle's medium (DMEM); H1299 cells were cultured in RPMI-1640, supplemented with 10% fetal bovine serum (FBS) at 37°C in a 5% CO_2_ humidified atmosphere.

### Plasmid construction and transfection

HPV16 E6 cDNA (p-EGFP-N1-HPV16E6 and p-EGFP-N1, gifts from Prof. Xu-Dong Tang, Institute of Biochemistry and Molecular Biology, Guangdong Medical College, China) was transfected into H1299 cell lines which expresses a relatively low level of HPV16 [22]. LKB1(pcDNA3-LKB1-His and pcDNA3-His, gifts from Prof. Xin Hou, College of Life Sciences, Inner Mongolia University, Huhhot, Inner Mongolia, China) was transfected into A549 cell lines which expresses a relatively low level of LKB1 [23]. The plasmids of mutants and empty were used as a negative control. Cells exposed to Lipofectamine^™^ 2000 or Oligofectamine^™^ alone served as mock transfection controls. The transfection efficiency was evaluated by observing green fluorescence under a fluorescence microscope and flow cytometric analysis (Epics-XL, Coulter, USA).

### Small interfering RNA (siRNA)

Short-interference RNAs (siRNAs) against E6, LKB1, and SP1 were purchased from RIBOBIO (Guangzhou, China). Scrambled siRNA (RIBOBIO, Guangzhou, China) was used as a nonspecific siRNA control. For the siRNA transfection, H1299 and A549 cells were seeded at 5x104 cells/35-mm well. Then 24 h later, the siRNA was transfected into the cells using Lipofectamine^™^ RNAiMAX reagent (Invitrogen, Carlsbad, CA, USA). After transfection, the cells were incubated for 48 h and subjected to various analyses.

### Western blot analysis

Total protein from cultured cells was extracted in cell lysis buffer (PIERCE, Rockford, IL) and quantified using the Bradford method. Fifty micrograms of protein were loaded and separated on SDS–PAGE (12%). After transferring to a polyvinylidene fluoride (PVDF) membrane (Millipore, Billerica, MA), the membrane was incubated overnight at 4°C with antibody against E6 (1:100; Bioss Biotechnology Co., Ltd, Beijing, China), LKB1 (1:1000 Cell Signaling, USA), SP1 (1:100; Bioss), hTERT(1:200, Bioss), or mouse monoclonal antibody against beta-actin or Rabbit monoclonal antibody against GAPDH (1:500; Santa Cruz Biotechnology, Santa Cruz, CA). After incubation with peroxidase-conjugated anti-mouse IgG (Santa Cruz Biotechnology) at 37°C for 2 h, bound proteins were visualized using ECL (Pierce) and detected using BioImaging Systems (UVP Inc., Upland, CA). The relative protein levels were calculated by normalizing to beta-actin protein as a loading reference.

### Real-time PCR

Total RNA was extracted from cells and isolated using an RNeasy RNA isolation kit (QIAGEN, Hilden, Germany). First-strand cDNA was synthesized from 1 μg of total RNA using ReverTra Ace (TOYOBO, Osaka, Japan). RT-PCR was performed using an aliquot of first-strand cDNA as a template under standard conditions with Taq DNA polymerase (QIAGEN). The real-time polymerase chain reaction (PCR) was carried out using SYBER GreenMaster Mix (Applied Biosystems, Tokyo, Japan). The primers used here were as follows:

E6-F: 5′-GTATGGAACAACATTAGAACAGCAA-3′,

E6-R: 5′-GTGGCTTTTGACAGTTAATACACC-3′;

LKB1-F: 5′- AGGGCCGTCAAGATCCTCAA -3′,

LKB1-R: 5′- GCATGCCACACACGCAGTA - 3′;

SP1-F: 5′- CTCAGTGCATTGGGTACTTCAGGA -3’,

SP1-R: 5′- CCACCTGCTGTGTCATCATGTATTC - 3′.

hTERT-F: 5’-GCGGAAGACAGTGGTGAACT -3′,

hTERT-R: 5’-AGCTGGAGTAGTCGCTCTGC-3′.

The amplified products of E6, LKB1, SP1, and hTERT were 80, 108, 146, and 141 bp in length, respectively.

### Assay of luciferase reporter

The H1299 cell line and A549 cell line seeded with 70% to 80% confluence in a 24-well plate one day before were co-transfected with the firefly luciferase reporter (0.2ug) along with the Renilla luciferase reporter (Promega Co) (0.02 ug) using Lipofectamine^™^ 3000 (Invitrogen, USA) for 6 h in transfection medium. After replacing the transfection medium with complete medium, the cells were incubated for 24 h. Cells lysates were analyzed for measurement of luciferase activities using the Dual-Luciferase Assay kit (Promega, Madison, WI, USA) according to the manufacturer's instructions. The pGMSP1-Lu was purchased from Genomeditech, China. Relative luciferase activity was calculated by normalizing the ratio of Firefly/Renilla luciferase to that of negative control-transfected cells.

### Statistical analyses

The SPSS 16.0 statistical software package (SPSS, Inc. Chicago, IL, USA) was used for all analyses. Student’s t-test was used to compare data from the densitometry analysis. The McNemar’s test was used to compare the mRNA expression of E6, LKB1, SP1, and hTERT in benign brushing cells and malignant ones. Spearman correlation analysis was used to determine the correlation among mRNA expression of E6, LKB1, SP1, and hTERT in malignant group. All data were presented as mean ± SD for in vitro experiments performed at least 3 times, ANOVA and LSD-test were employed for statistical analysis. The level of statistical significance was set at *P* < 0.05.

## Results

The qRT-PCR results of brushing cells from patients with benign or malignant lung diseases, including AC and SCC, were presented in Table 1 and S1 Table.

**Table 1:** The mRNA of E6, LKB1, Sp1 and hTERT with qRT-PCR in bronchial brushing cells of patients with benign and malignant lung lesions (Mean ± SEM).

Histology	n	E6	LKB1	SP1	hTERT
Benign	68	0.010±0.003	0.481±0.063	0.019±0.001	0.042±0.006
Inflammation	58	0.01±0.004	0.481±0.067	0.018±0.001	0.039±0.006
Tuberculosis	10	0.009±0.001	0.478±0.181	0.021±0.004	0.058±0.022
Malignant	106	0.020±0.001*	0.007±0.001*	0.094±0.007*	1.089±0.126*
SCC	86	0.016±0.001*	0.007±0.001*	0.072±0.006*	0.608±0.085*
AC	20	0.036±0.004*	0.010±0.001*	0.189±0.004*	3.157±0.223*

qRT-PCR, quantitative real-time reverse transcriptase-polymerase chain reaction; SCC, squamous cell carcinoma; AC, adenocarcinoma; E6, Human papillomaviruses 16 E6; LKB1, Liver kinase B1; SP1, specificity protein 1; hTERT, human telomerase reverse transcriptase.

* *P* < 0.01 as compared to benign

E6, SP1, and hTERT mRNA were significantly increased in the malignant group than the benign group, whereas LKB1 mRNA was significantly decreased in the malignant group (*P* < 0.01). In the malignant group, SP1 mRNA was strongly positively correlated with E6 and hTERT mRNA (*P* < 0.01), whereas LKB1 mRNA was negatively correlated with E6, SP1, and hTERT mRNA (*P* < 0.01).

**Fig 1:**
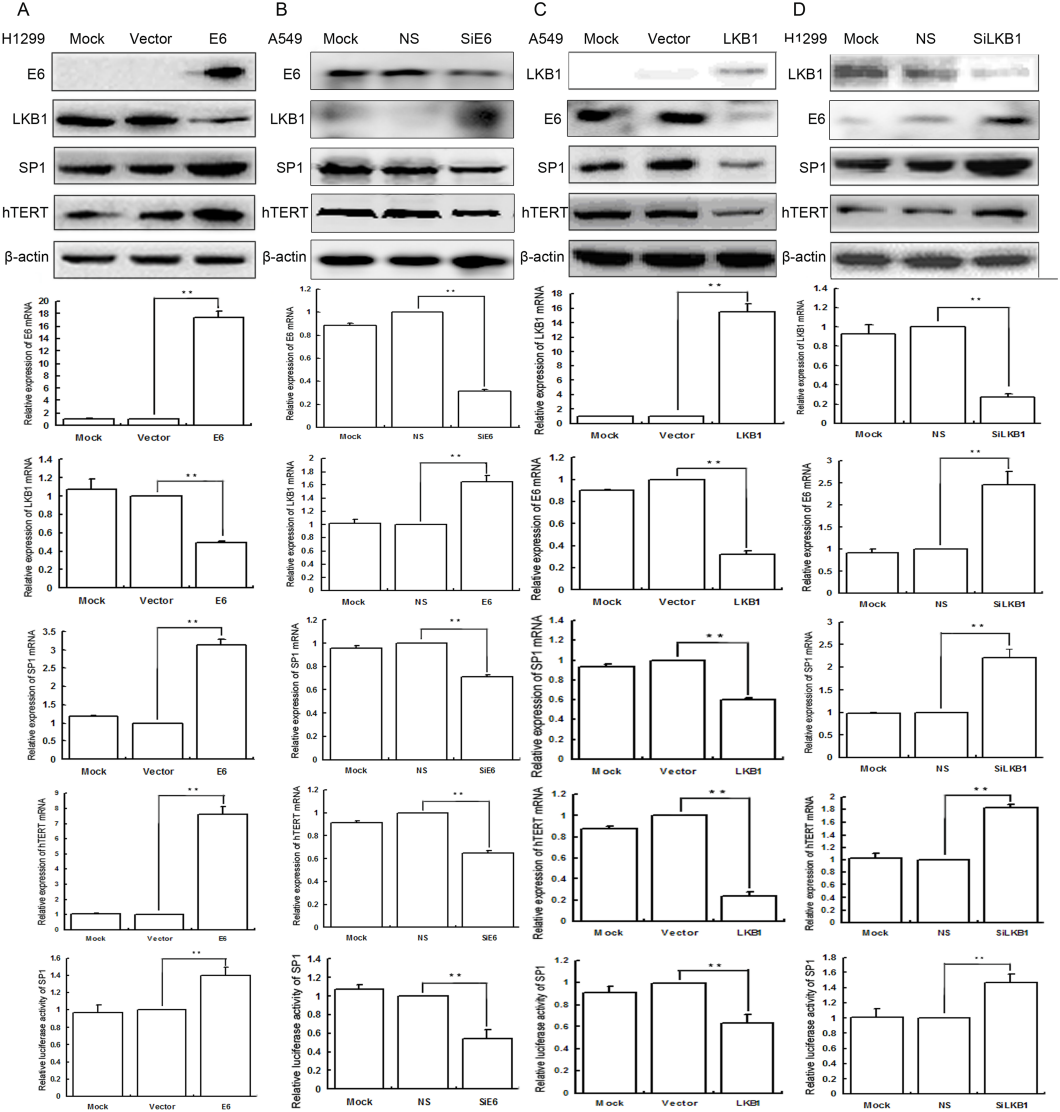
A Overexpression of E6, SP1, and hTERT but low expression of LKB1 was in H1299 cells. Mock mock transfection, Vector empty vector. B Low expression of E6 SP1, and hTERT but overexpression of LKB1 was in A549 cells. Mock mock-specific siRNA; NS nonspecific siRNA, SiE6 E6-specific siRNA. C Overexpression of LKB1 but low expression of E6, SP1, and hTERT was in A549 cells. Mock mock transfection, Vector empty vector. D Low expression of LKB1 but overexpression of E6, SP1, and hTERT was in H1299 cells. Mock mock-specific siRNA; NS nonspecific siRNA, SiLKB1 LKB1-specific siRNA. Detection the expression of both protein and mRNA of E6, LKB1, SP1, and hTERT was in lung cancer cells as well as control cells by Western blot and qRT-PCR. (** *P* < 0.01). β-actin served as internal controls.

Overexpression of E6 significantly downregulated the expression of both protein and mRNA of LKB1, but upregulated the expression of both protein and mRNA of SP1 and hTERT, as well as the transcriptional activity of SP1 in H1299 cells; the results were presented in Fig 1A, S1 Fig and S2 Table.

Transiently transfected pEGFP-N1-E6 into the low expression H1299 cell lines, and using E6 empty vector and mock transfection served as controls. The results showed that overexpression of E6 significantly downregulated the expression of both protein and mRNA of LKB1, but upregulated the expression of both protein and mRNA of SP1 and hTERT as well as the transcriptional activity of SP1.

Inhibition of E6 upregulated the expression of both protein and mRNA of LKB1, but downregulated the expression of both protein and mRNA of SP1 and hTERT, as well as the transcriptional activity of SP1 in A549 cells; the results are presented in Fig 1B, S2 Fig and S2 Table.

In order to further verify the regulatory roles of E6 on LKB1, SP1, and hTERT, we applied E6-specific siRNA to knockdown the expression of E6 in A549 cell lines, and using E6-nonspecific siRNA and mock specific siRNA served as controls. The results indicated that inhibition of E6 upregulated the expression of both protein and mRNA of LKB1, but downregulated the expression of both protein and mRNA of SP1 and hTERT as well as the transcriptional activity of SP1 in A549 cells. E6-nonspecific siRNA and mock specific siRNA showed minimal or no change.

Overexpression of LKB1 downregulated the expression of both protein and mRNA of SP1, hTERT, and E6, as well as the transcriptional activity of SP1 in A549 cells; the results were presented in Fig 1C, S3 Fig and S3 Table.

We transiently transfected pcDNA3-LKB1-His into the low expression A549 cell lines, and using LKB1 empty vector and mock transfection served as controls. The results showed that overexpression of LKB1 downregulated the expression of both protein and mRNA of SP1, hTERT, and E6, as well as the transcriptional activity of SP1.

Inhibition of LKB1 upregulated the expression of both protein and mRNA of SP1, hTERT, and E6, as well as the transcriptional activity of SP1 in H1299 cells; the results were presented in Fig 1D, S4 Fig and S3 Table.

In order to further verify the regulatory roles of LKB1 on SP1, hTERT, and E6, we used LKB1-specific siRNA to knockdown the expression of LKB1 in H1299 cell lines, and using LKB1-nonspecific siRNA and mock specific siRNA served as controls. The results showed that inhibition of LKB1 upregulated the expression of both protein and mRNA of SP1, hTERT, and E6, as well as the transcriptional activity of SP1 in H1299 cells. LKB1-nonspecific siRNA and mock specific siRNA showed minimal or no change.

**Fig 2:**
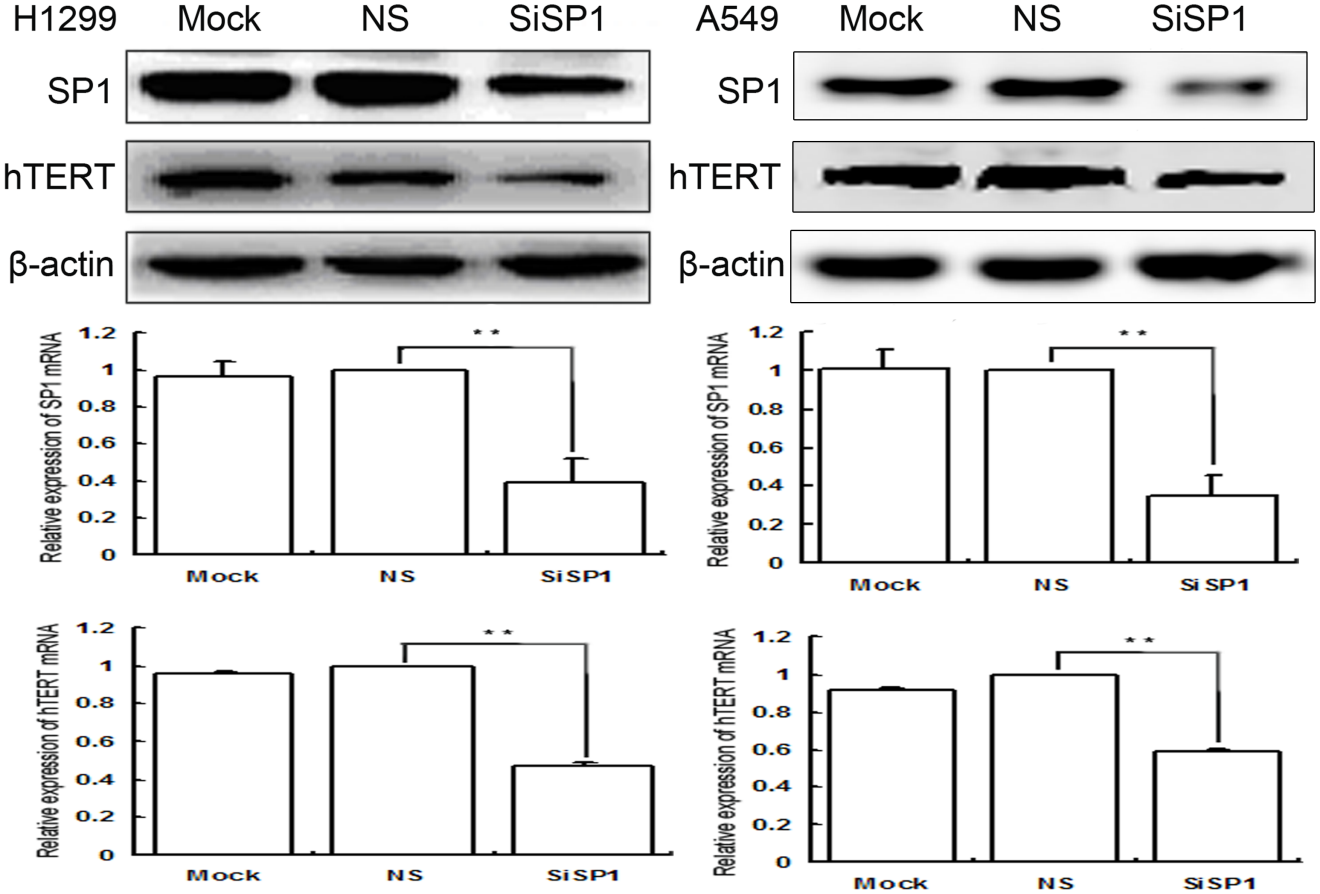
Low expression of SP1 and hTERT was in H1299 and A549 cells. Detection the expression of both protein and mRNA of them was in lung cancer cells as well as in control cells by Western blot and qRT-PCR. Mock mock-specific siRNA, NS nonspecific siRNA, SiSP1 SP1-specific siRNA (** *P* < 0.01). β-actin served as internal controls.

Inhibition of SP1 down-regulated the expression of both protein and mRNA of hTERT in H1299 and A549 cells; the results were presented in Fig 2, S5 and S6 Figs and S4 Table.

In order to further verify the regulatory roles of E6 on hTERT was mediated by SP1, we utilized SP1-specific siRNA to knockdown the expression of SP1 in both H1299 and A549 cell lines, SP1-nonspecific siRNA, and mock specific siRNA served as controls. The results showed that inhibition of SP1 down-regulated the expression of both protein and mRNA of hTERT.

## Discussion

Human papillomaviruses are small oncogenic DNA viruses of which more than 200 types have been identified to date [24]. A causative relationship between high risk HPV infection and human cervical cancers or a subset of head and neck squamous cell carcinomas has been defined [25]. In recent years, more and more evidence had also implicated the association of high-risk HPV infection and the occurrence and progression of non-small cell lung cancer [3–9, 26]. Inactivation of LKB1 is a common event in adenocarcinomas of the lung [27], and long-term persistent infection of high-risk HPV is a necessary condition; otherwise the body of a transient infection will be automatically outcome. However, the underlying molecular mechanism of this phenomenon is unclear at present. The current study found that E6 had a significant inhibitory effect on LKB1, while LKB1 had a significant inhibitory effect on E6 as well. Thus, our results indicated that LKB1 acted as a safeguard against long-term persistent infection of high-risk HPV. Furthermore, long-term persistent infection of high-risk HPV 16 E6 caused LKB1 accumulated mutations and LKB1 loss that may be one of the main cause of lung cancer occurrence.

Bronchial brushing is an important method in the cytological diagnosis of lung cancer during the same bronchoscopy [28, 29], and it is also an important supplement to biopsy. A combination of brushing and forceps biopsy is considered to be an essential step in the diagnosis of bronchoscopically visible lung cancer [30, 31]. Recently, we have used RT-PCR for detecting cancer cells in bronchial brushing specimens of patients with lung cancer, and demonstrated that this method is more sensitive than conventional smear preparations [32–34]. In the present study, the expression of E6, SP1 and hTERT mRNAs were increased in the lung cancer group than in the benign group by qRT-PCR (*P* < 0.01), conversely, LKB1 mRNA was decreased in the lung cancer (*P* < 0.01). Furthermore, LKB1 was significantly negative correlated with E6 and SP1 in the malignant group (*P* < 0.01), but SP1 was significantly correlated with hTERT in the malignant group (*P* < 0.01). Our results indicated that LKB1 was likely to have gene regulatory relations with E6 and SP1, and SP1 regulatory the expression of hTERT.

In order to investigate the roles of LKB1 on regulating the expression of SP1 and hTERT in lung cancer cells, we transiently transfected pcDNA3-LKB1-His into the low expression NSCLC cell lines, A549 cells. The results showed that overexpression of LKB1 significantly downregulated the expression of both protein and mRNA of SP1 and hTERT as well as the transcriptional activity of SP1. Furthermore we employed siRNA to knockdown the expression of LKB1 in H1299 cell lines. The results showed that inhibition of LKB1 significantly upregulated the expression of both protein and mRNA of SP1 and hTERT as well as the transcriptional activity of SP1. These results demonstrated SP1 and hTERT are down-stream effectors of LKB1. In order to further verify the regulation relationship between SP1 and hTERT, we used siRNA to knockdown the expression of SP1 in both H1299 and A549 cell lines. The results showed that inhibition of SP1 significantly down-regulated the expression of both protein and mRNA of hTERT. This was in agreement with a recently published report by Long et al [21]. The above results suggest that SP1 may be a direct target gene of LKB1, and that the inhibition of SP1 expression by LKB1 may be accomplished by the phosphorylation of SP1. This conjecture and the specific phosphorylation sites of SP1 need to be further studied in the future.

In HPV related lung cancer, E6 upregulates hTERT [34], but the underlying molecular mechanism is not clear. Our study found that E6 and LKB1 have inhibitory effect on each other. Long-term persistent infection of high-risk HPV16 E6 caused LKB1 loss, and E6 upregulated the expression of both protein and mRNA of hTERT in lung cancer cells mediated by SP1. Cheng et al. used the interference technique to knock down E6 in lung cancer cells, and the results showed that there was no change in SP1 protein expression. However, we used the transfection and interference technology to regulate the E6 in lung cancer cells, the results showed that the expression of SP1 protein was associated with the change of E6. [35].

The activation of telomerase, a critical step in the process of human cell immortality and malignant transformation, had been widely observed in human malignant tumors. Telomerase activity is closely related to the expression of hTERT. Thus, the expression of hTERT protein, a prerequisite for obtaining telomerase activity, had been shown to be a major determinant of telomerase activity control [36,37].

In conclusion we demonstrated that the expression of E6, SP1, and hTERT mRNAs were increased in brushing cells of patients with lung cancer compared with benign disease by qRT-PCR (*P* < 0.01). The correlation comparison indicated that E6, SP1, and hTERT with positive, but LKB1 with negative correlation (*P* < 0.01). We had demonstrated for the first time that E6 upregulation of hTERT by downregulated LKB1 and upregulated SP1 in lung cancer cells. These results indicated that E6 played a predominant role in the regulation of telomerase activation and may be a valuable therapeutic targets for HPV-related cancer.

## Supporting information

S1 Fig Overexpression of E6, SP1, and hTERT but low expression of LKB1 was in H1299 cells. Transiently transfected pEGFP-N1-E6 into the low expression H1299 cell lines.
(JPG)

S2 Fig Low expression of E6 SP1, and hTERT but overexpression of LKB1 was in A549 cells. E6-specific siRNA was used to knockdown the expression of E6 in A549 cell lines.
(TIF)

S3 Fig Overexpression of LKB1 but low expression of E6, SP1, and hTERT was in A549 cells. Transiently transfected pcDNA3-LKB1-His into the low expression A549 cell lines.
(TIF)

S4 Fig Low expression of LKB1 but overexpression of E6, SP1, and hTERT was in H1299 cells. LKB1-specific siRNA was used to knockdown the expression of LKB1 in H1299 cell lines.
(JPG)

S5 Fig Low expression of SP1 and hTERT was in H1299 cells. SP1-specific siRNA was used to knockdown the expression of SP1 in H1299 cell lines.
(JPG)

S6 Fig Low expression of SP1 and hTERT was in A549 cells. SP1-specific siRNA was used to knockdown the expression of SP1 in A549 cell lines.
(TIF)

S1 Table The mRNAs of E6, LKB1, SP1 and hTERT with qRT-PCR in bronchial brushing cells of patients with inflammation. (DOC)

S2 Table The qRT-PCR results for E6 by transfection and SiRNAs in H1299 and A549 cell lines. (DOC)

S3 Table The qRT-PCR results for the LKB1 by Transfection and SiRNAs in A549 and H1299 cell lines. (DOC)

S4 Table The qRT-PCR results for the SP1 by SiRNAs in both A549 and H1299 cell lines. (DOC)

## Abbreviations

HPV: Human papillomaviruses
hTERT: human telomerase reverse transcriptase
LKB1: Liver kinase B1
NSCLC: non-small cell lung carcinoma
qRT-PCR: quantitative real-time reverse transcriptase-polymerase chain reaction
SP1: specificity protein 1

## References

[1] Hawley-Nelson P, Vousden KH, Hubbert NL, Lowy DR, Schiller JT. HPV16 E6 and E7 proteins cooperate to immortalize human foreskin keratinocytes. EMBO J. 1989; 8(12): 3905–10. 2555178 10.1002/j.1460-2075.1989.tb08570.x PMC402081

[2] Syrjänen KJ. Condylomatous changes in neoplastic bronchial epithelium. Report of a case.Respiration. 1979; 38(5): 299–304. 538337 10.1159/000194095

[3] Ciotti M, Giuliani L, Ambrogi V, Ronci C, Benedetto A, Mineo TC, et al Detection and expression of human papillomavirus oncogenes in non-small cell lung cancer.Oncol Rep. 2006; 16(1): 183–9. 16786144

[4] Wang Y, Wang A, Jiang R, Pan H, Huang B, Lu Y, Wu C. Human papillomavirus type 16 and 18 infection is associated with lung cancer patients from the central part of China. Oncol Rep. 2008; 20(2): 333–9. 18636194

[5] Srinivasan M, Taioli E, Ragin CC. Human papillomavirus type 16 and 18 in primary lung cancers—a meta-analysis. Carcinogenesis. 2009; 30(10): 1722–8. doi: 10.1093/carcin/bgp177 19620233 10.1093/carcin/bgp177 PMC2764507

[6] Baba M, Castillo A, Koriyama C, Yanagi M, Matsumoto H, Natsugoe S, et al Human papillomavirus is frequently detected in gefitinib-responsive lung adenocarcinomas. Oncol Rep. 2010; 23(4): 1085–92. 20204295 10.3892/or_00000736

[7] Aguayo F, Anwar M, Koriyama C, Castillo A, Sun Q, Morewaya J, et al Human papillomavirus-16 presence and physical status in lung carcinomas from Asia. Infect Agent Cancer. 2010; 5: 20 doi: 10.1186/1750-9378-5-20 21080966 10.1186/1750-9378-5-20 PMC2994794

[8] Zhang J, Wang T, Han M, Yang ZH, Liu LX, Chen Y, et al Variation of human papillomavirus 16 in cervical and lung cancers in Sichuan, China. Acta Virol. 2010; 54(4): 247–53. 21175246 10.4149/av_2010_04_247

[9] Joh J, Jenson AB, Moore GD, Rezazedeh A, Slone SP, Ghim SJ, et al Human papillomavirus (HPV) and Merkel cell polyomavirus (MCPyV) in non small cell lung cancer. Exp Mol Pathol. 2010; 89(3): 222–6. doi: 10.1016/j.yexmp.2010.08.001 20699096 10.1016/j.yexmp.2010.08.001

[10] Zhang E, Feng X, Liu F, Zhang P, Liang J, Tang X. Roles of PI3K/Akt and c-Jun signaling pathways in human papillomavirus type 16 oncoprotein-induced HIF-1α, VEGF, and IL-8 expression and in vitro angiogenesis in non-small cell lung cancercells. PLoS One. 2014; 9(7): e103440 doi: 10.1371/journal.pone.0103440 25058399 10.1371/journal.pone.0103440 PMC4110025

[11] Mack HI, Munger K. The LKB1 tumor suppressor differentially affects anchorage independent growth of HPV positive cervical cancer cell lines. Virology. 2013; 446(1–2): 9–16. doi: 10.1016/j.virol.2013.07.009 24074562 10.1016/j.virol.2013.07.009 PMC3843939

[12] Tsai LH, Wu JY, Cheng YW, Chen CY, Sheu GT, Wu TC, et al The MZF1/c-MYC axis mediates lung adenocarcinoma progression caused by wild-type lkb1 loss. Oncogene. 2015; 34(13): 1641–9. doi: 10.1038/onc.2014.118 24793789 10.1038/onc.2014.118

[13] Whang YM, Park SI, Trenary IA, Egnatchik RA, Fessel JP, Kaufman JM, et al LKB1 deficiency enhances sensitivity to energetic stress induced by erlotinib treatment in non-small-cell lung cancer (NSCLC) cells. Oncogene. 2016; 35(7): 856–66. doi: 10.1038/onc.2015.140 26119936 10.1038/onc.2015.140 PMC4486321

[14] Hemminki A, Markie D, Tomlinson I, Avizienyte E, Roth S, Loukola A, et al A serine/threonine kinase gene defective in Peutz-Jeghers syndrome. Nature. 1998; 391(6663): 184–7. doi: 10.1038/34432 9428765 10.1038/34432

[15] Mehenni H, Resta N, Park JG, Miyaki M, Guanti G, Costanza MC. Cancer risks in LKB1 germline mutation carriers. Gut. 2006; 55(7): 984–90. doi: 10.1136/gut.2005.082990 16407375 10.1136/gut.2005.082990 PMC1856321

[16] Jimenez AI, Fernandez P, Dominguez O, Dopazo A, Sanchez-Cespedes M. Growth and molecular profile of lung cancer cells expressing ectopic LKB1: down-regulation of the phosphatidylinositol 3'-phosphate kinase/PTEN pathway. Cancer Res. 2003; 63(6): 1382–8. 12649203

[17] Tsai LH, Chen PM, Cheng YW, Chen CY, Sheu GT, Wu TC, et al LKB1 loss by alteration of the NKX2-1/p53 pathway promotes tumor malignancy and predicts poor survival and relapse in lung adenocarcinomas. Oncogene. 2014; 33(29): 3851–60. doi: 10.1038/onc.2013.353 23995788 10.1038/onc.2013.353

[18] Liang X, Li ZL, Jiang LL, Guo QQ, Liu MJ, Nan KJ. Suppression of lung cancer cell invasion by LKB1 is due to the downregulation of tissue factor and vascular endothelial growth factor, partly dependent on SP1. Int J Oncol. 2014; 44(6): 1989–97. doi: 10.3892/ijo.2014.2351 24647869 10.3892/ijo.2014.2351

[19] Cha N, Lv M, Zhao YJ, Yang D, Wang EH, Wu GP. Diagnostic utility of VEGF mRNA and SP1 mRNA expression in bronchial cells of patients with lung cancer. Respirology. 2014; 19(4): 544–8. doi: 10.1111/resp.12272 24661424 10.1111/resp.12272

[20] Hedrick E, Cheng Y, Jin UH, Kim K, Safe S. Specificity protein (Sp) transcription factors SP1, Sp3 and Sp4 are non-oncogene addiction genes in cancer cells. Oncotarget. 2016; 7(16): 22245–56. doi: 10.18632/oncotarget.7925 26967243 10.18632/oncotarget.7925 PMC5008359

[21] Long C, Wang J, Guo W, Wang H, Wang C, Liu Y, et al Triptolide inhibits transcription of hTERT through down-regulation of transcription factor specificity protein 1 in primary effusion lymphoma cells. Biochem Biophys Res Commun. 2016; 469(1): 87–93 doi: 10.1016/j.bbrc.2015.11.076 26631963 10.1016/j.bbrc.2015.11.076

[22] Fan R, Hou WJ, Zhao YJ, Liu SL, Qiu XS, Wang EH, et al Overexpression of HPV16 E6/E7 mediated HIF-1α upregulation of GLUT1 expression in lung cancer cells. Tumour Biol. 2016; 37(4): 4655–63. doi: 10.1007/s13277-015-4221-5 26508030 10.1007/s13277-015-4221-5

[23] Hou X, Liu JE, Liu W, Liu CY, Liu ZY, Sun ZY. A new role of NUAK1: directly phosphorylating p53 and regulating cell proliferation. Oncogene. 2011; 30(26): 2933–42. doi: 10.1038/onc.2011.19 21317932 10.1038/onc.2011.19

[24] Haedicke J, Iftner T. Human papillomaviruses and cancer. Radiother Oncol. 2013; 108(3): 397–402. doi: 10.1016/j.radonc.2013.06.004 23830197 10.1016/j.radonc.2013.06.004

[25] Hellner K, Münger K. Human papillomaviruses as therapeutic targets in human cancer. J Clin Oncol. 2011; 29(13): 1785–94. doi: 10.1200/JCO.2010.28.2186 21220591 10.1200/JCO.2010.28.2186 PMC3675666

[26] Li G, He L, Zhang E, Shi J, Zhang Q, Le AD, et al Overexpression of human papillomavirus (HPV) type 16 oncoproteins promotes angiogenesis via enhancing HIF-1α and VEGF expression in non-small cell lung cancer cells. Cancer Lett. 2011; 311(2): 160–70. doi: 10.1016/j.canlet.2011.07.012 21868151 10.1016/j.canlet.2011.07.012

[27] Sanchez-Cespedes M, Parrella P, Esteller M, Nomoto S, Trink B, Engles JM, et al Inactivation of LKB1/STK11 is a common event in adenocarcinomas of the lung. Cancer Res. 2002; 62(13): 3659–62. 12097271

[28] van der Drift MA, van der Wilt GJ, Thunnissen FB, Janssen JP. A prospective study of the timing and cost-effectiveness of bronchial washing during bronchoscopy for pulmonary malignant tumors. Chest 2005; 128(1): 394–400. doi: 10.1378/chest.128.1.394 16002962 10.1378/chest.128.1.394

[29] Mak VH, Johnston ID, Hetzel MR, Grubb C. Value of washings and brushings at fibreoptic bronchoscopy in the diagnosis of lung cancer. Thorax 1990; 45(5): 373–6. 2200159 10.1136/thx.45.5.373 PMC462478

[30] Matsuda M, Horai T, Nakamura S, Nishio H, Sakuma T, Ikegami H, et al Bronchial brushing and bronchial biopsy: comparison of diagnostic accuracy and cell typing reliability in lung cancer. Thorax 1986; 41(6): 475–8. 3024348 10.1136/thx.41.6.475 PMC460368

[31] Karahalli E, Yilmaz A, Türker H, Ozvaran K. Usefulness of various diagnostic techniques during fiberoptic bronchoscopy for endoscopically visible lung cancer: should cytologic examinations be performed routinely? Respiration 2001; 68(6):611–4. 11786717 10.1159/000050581

[32] Lv M, Wu MZ, Zhao YJ, Yang DI, Wang EH, Wu GP. Expression and clinical significance of LUNX mRNA in bronchial brushing specimens from patients with and without lung cancer. Respirology 2011; 16(7): 1076–80. doi: 10.1111/j.1440-1843.2011.02008.x 21651644 10.1111/j.1440-1843.2011.02008.x

[33] Lv M, Fan YB, Zhao YJ, Wang TY, Wu GP. Expression and clinical significance of glucose transporter 1 mRNA in bronchial brushing specimens from patients with and without lung cancer. Cytopathology 2012; 23(2): 108–13. doi: 10.1111/j.1365-2303.2010.00846.x 21294792 10.1111/j.1365-2303.2010.00846.x

[34] Cha N, Lv M, Zhao YJ, Yang D, Wang EH, Wu GP. Diagnostic utility of VEGF mRNA and SP1 mRNA expression in bronchial cells of patients with lung cancer. Respirology. 2014; 19(4): 544–8. doi: 10.1111/resp.12272 24661424 10.1111/resp.12272

[35] Cheng YW, Wu TC, Chen CY, Chou MC, Ko JL, Lee H. Human telomerase reverse transcriptase activated by E6 oncoprotein is required for human papillomavirus—16/18—infected lung tumorigenesis. Clin Cancer Res. 2008; 14(22): 7173–9. doi: 10.1158/1078-0432.CCR-08-0850 19010833 10.1158/1078-0432.CCR-08-0850

[36] Nakamura TM, Morin GB, Chapman KB, Weinrich SL, Andrews WH, Lingner J, et al Telomerase catalytic subunit homologs from fission yeast and human. Science. 1997; 277(5328): 955–9. 9252327 10.1126/science.277.5328.955

[37] Meyerson M, Counter CM, Eaton EN, Ellisen LW, Steiner P, Caddle SD, et al hEST 2, the putative human telomerase catalytic subunit gene, is up-regulated in tumor cells and during immortalization. Cell. 1997; 90(4): 785–95. 9288757 10.1016/s0092-8674(00)80538-3

