# Supplementary figures and images for: Long-term persistent infection of HPV 16 E6 up-regulate SP1 and hTERT by inhibiting LKB1 in lung cancer cells
Source: PLoS One. 2017 Aug 16;12(8):e0182775. doi: 10.1371/journal.pone.0182775 (PMC5558957; doi:10.1371/journal.pone.0182775)

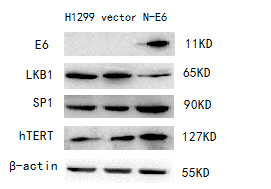

Supplement: S1 Fig — Transiently transfected pEGFP-N1-E6 into the low expression H1299 cell lines. (JPG) [file pone.0182775.s001.jpg]

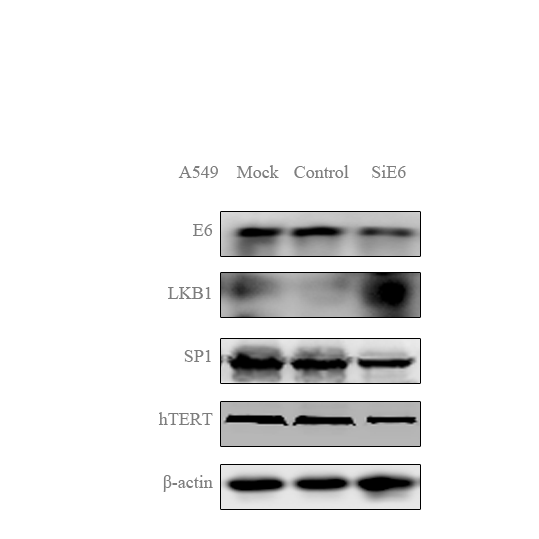

Supplement: S2 Fig — E6-specific siRNA was used to knockdown the expression of E6 in A549 cell lines. (TIF) [file pone.0182775.s002.tif]

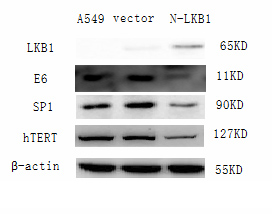

Supplement: S3 Fig — Transiently transfected pcDNA3-LKB1-His into the low expression A549 cell lines. (TIF) [file pone.0182775.s003.tif]

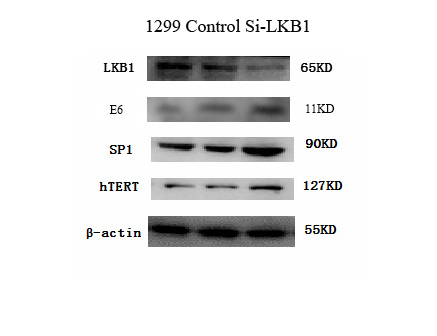

Supplement: S4 Fig — LKB1-specific siRNA was used to knockdown the expression of LKB1 in H1299 cell lines. (JPG) [file pone.0182775.s004.jpg]

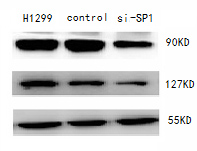

Supplement: S5 Fig — SP1-specific siRNA was used to knockdown the expression of SP1 in H1299 cell lines. (JPG) [file pone.0182775.s005.jpg]

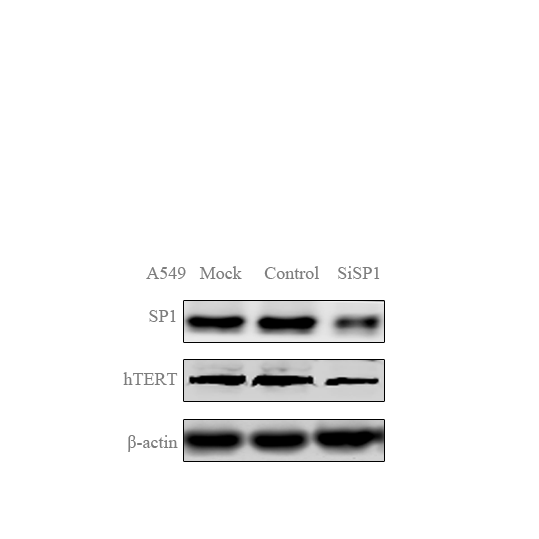

Supplement: S6 Fig — SP1-specific siRNA was used to knockdown the expression of SP1 in A549 cell lines. (TIF) [file pone.0182775.s006.tif]
