# Supplementary material for: Long-term persistent infection of HPV 16 E6 up-regulate SP1 and hTERT by inhibiting LKB1 in lung cancer cells
Source: PLoS One. 2017 Aug 16;12(8):e0182775. doi: 10.1371/journal.pone.0182775 (PMC5558957; doi:10.1371/journal.pone.0182775)
Supplement: S1 Table — (DOC) [file pone.0182775.s007.doc]

S1-Table. The mRNAs of E6, LKB1, SP1 and hTERT with qRT-PCR in bronchial brushing cells of patients with Inflammation

| Specimen | Age | Sex | Pathologic Diagnosis | E6 | LKB1 | SP1 | hTERT |
| --- | --- | --- | --- | --- | --- | --- | --- |
| 1 | 59 | Female | Local dysplasia | 0.01501 | 0.67851 | 0.02037 | 0.07280 |
| 2 | 49 | male | Local dysplasia | 0.01297 | 0.54896 | 0.01853 | 0.02362 |
| 3 | 63 | male | Local dysplasia | 0.01278 | 0.04067 | 0.04299 | 0.10584 |
| 4 | 75 | male | Inflammation | 0.00955 | 0.21476 | 0.02304 | 0.00352 |
| 5 | 69 | male | Inflammation | 0.00526 | 0.04152 | 0.00964 | 0.00272 |
| 6 | 63 | male | Inflammation | 0.00300 | 1.16254 | 0.02256 | 0.04653 |
| 7 | 48 | male | Inflammation | 0.00292 | 0.43158 | 0.00503 | 0.05268 |
| 8 | 59 | Female | Inflammation | 0.01610 | 0.03983 | 0.00533 | 0.00537 |
| 9 | 58 | male | Inflammation | 0.01311 | 0.75627 | 0.01765 | 0.00240 |
| 10 | 59 | male | Inflammation | 0.13830 | 0.16348 | 0.01987 | 0.07642 |
| 11 | 57 | male | Inflammation | 0.00170 | 0.18452 | 0.02320 | 0.00233 |
| 12 | 24 | male | Inflammation | 0.01286 | 0.03615 | 0.00652 | 0.00251 |
| 13 | 61 | male | Inflammation | 0.00191 | 0.03640 | 0.01346 | 0.03928 |
| 14 | 64 | male | Inflammation | 0.16724 | 0.47852 | 0.02758 | 0.02574 |
| 15 | 39 | male | Inflammation | 0.01103 | 0.85263 | 0.01379 | 0.00427 |
| 16 | 61 | male | Inflammation | 0.01137 | 0.03025 | 0.00865 | 0.00662 |
| 17 | 43 | male | Inflammation | 0.00038 | 0.19235 | 0.02712 | 0.00792 |
| 18 | 57 | male | Inflammation | 0.01512 | 1.20124 | 0.02510 | 0.07748 |
| 19 | 49 | Female | Inflammation | 0.00769 | 0.02836 | 0.01152 | 0.02759 |
| 20 | 32 | Female | Inflammation | 0.00040 | 0.05219 | 0.01733 | 0.02628 |

| Specimen | Age | Sex | Pathologic Diagnosis | E6 | LKB1 | SP1 | hTERT |
| --- | --- | --- | --- | --- | --- | --- | --- |
| 21 | 67 | male | Inflammation | 0.00046 | 0.02896 | 0.01370 | 0.04986 |
| 22 | 56 | Female | Inflammation | 0.00630 | 0.07365 | 0.02366 | 0.07663 |
| 23 | 61 | Female | Inflammation | 0.00894 | 0.03365 | 0.03665 | 0.00455 |
| 24 | 64 | male | Inflammation | 0.00139 | 1.63252 | 0.01220 | 0.02164 |
| 25 | 62 | male | Inflammation | 0.01024 | 0.09479 | 0.00596 | 0.06856 |
| 26 | 38 | Female | Inflammation | 0.00131 | 0.08538 | 0.02538 | 0.00792 |
| 27 | 66 | male | Inflammation | 0.01433 | 0.01734 | 0.02422 | 0.29645 |
| 28 | 65 | male | Inflammation | 0.00910 | 1.59861 | 0.01067 | 0.00377 |
| 29 | 76 | male | Inflammation | 0.00102 | 1.17024 | 0.02102 | 0.00861 |
| 30 | 53 | Female | Inflammation | 0.00258 | 0.02739 | 0.02636 | 0.00923 |
| 31 | 28 | male | Inflammation | 0.01314 | 0.06896 | 0.00921 | 0.04299 |
| 32 | 48 | Female | Inflammation | 0.00244 | 0.02856 | 0.03280 | 0.00203 |
| 33 | 61 | Female | Inflammation | 0.00035 | 1.15348 | 0.01402 | 0.03358 |
| 34 | 48 | male | Inflammation | 0.00923 | 0.62146 | 0.02537 | 0.03615 |
| 35 | 27 | Female | Inflammation | 0.00103 | 1.00321 | 0.00699 | 0.06722 |
| 36 | 50 | Female | Inflammation | 0.00099 | 0.71457 | 0.00521 | 0.02486 |
| 37 | 49 | male | Inflammation | 0.00161 | 0.03928 | 0.01332 | 0.02956 |
| 38 | 72 | Female | Inflammation | 0.00212 | 0.13490 | 0.00786 | 0.02943 |
| 39 | 41 | male | Inflammation | 0.00091 | 0.07911 | 0.00481 | 0.00269 |
| 40 | 47 | Female | Inflammation | 0.00118 | 0.02574 | 0.00491 | 0.00770 |
| Specimen | Age | Sex | Pathologic Diagnosis | E6 | LKB1 | SP1 | hTERT |
| 41 | 50 | male | Inflammation | 0.00121 | 0.52684 | 0.00942 | 0.19863 |
| 42 | 49 | male | Inflammation | 0.00126 | 0.01698 | 0.02574 | 0.07690 |
| 43 | 59 | Female | Inflammation | 0.00252 | 1.39841 | 0.03983 | 0.00375 |
| 44 | 75 | male | Inflammation | 0.00115 | 0.92315 | 0.01824 | 0.06561 |
| 45 | 63 | Female | Inflammation | 0.00130 | 1.45621 | 0.01360 | 0.05642 |
| 46 | 30 | male | Inflammation | 0.00508 | 0.82932 | 0.02351 | 0.00481 |
| 47 | 38 | Female | Inflammation | 0.00134 | 1.14841 | 0.03019 | 0.04025 |
| 48 | 58 | male | Inflammation | 0.00028 | 0.15604 | 0.00756 | 0.00803 |
| 49 | 60 | male | Inflammation | 0.00098 | 0.34153 | 0.02288 | 0.00624 |
| 50 | 64 | Female | Inflammation | 0.00039 | 0.04671 | 0.01618 | 0.05872 |
| 51 | 57 | Female | Inflammation | 0.00020 | 0.26352 | 0.02977 | 0.07413 |
| 52 | 31 | Female | Inflammation | 0.00032 | 0.19478 | 0.01698 | 0.00963 |
| 53 | 42 | Female | Inflammation | 0.00261 | 1.23516 | 0.01398 | 0.04303 |
| 54 | 54 | Female | Inflammation | 0.00035 | 1.55248 | 0.02936 | 0.06756 |
| 55 | 48 | Female | Inflammation | 0.00017 | 0.38746 | 0.02386 | 0.03280 |
| 56 | 59 | male | Inflammation | 0.01506 | 1.14642 | 0.01427 | 0.03756 |
| 57 | 59 | Female | Inflammation | 0.00027 | 0.30255 | 0.01686 | 0.00832 |
| 58 | 58 | Female | Inflammation | 0.00099 | 0.17237 | 0.02457 | 0.03235 |

2.The mRNAs of E6, LKB1, Sp1 and hTERT with qRT-PCR in bronchial brushing cells of patients with Tuberculosis

| Specimen | Age | Sex | Pathologic Diagnosis | E6 | LKB1 | SP1 | hTERT |
| --- | --- | --- | --- | --- | --- | --- | --- |
| 1 | 45 | Female | Tuberculosis | 0.00824 | 0.11237 | 0.02856 | 0.00501 |
| 2 | 57 | Female | Tuberculosis | 0.00739 | 0.02062 | 0.01652 | 0.06561 |
| 3 | 70 | Female | Tuberculosis | 0.00174 | 0.01871 | 0.03768 | 0.00303 |
| 4 | 21 | male | Tuberculosis | 0.00820 | 1.36215 | 0.00737 | 0.01209 |
| 5 | 72 | Female | Tuberculosis | 0.01303 | 1.17548 | 0.04210 | 0.07326 |
| 6 | 71 | Female | Tuberculosis | 0.00630 | 0.02164 | 0.00852 | 0.07032 |
| 7 | 36 | male | Tuberculosis | 0.01252 | 0.03205 | 0.01204 | 0.00897 |
| 8 | 38 | Female | Tuberculosis | 0.01629 | 1.03013 | 0.02456 | 0.23685 |
| 9 | 55 | Female | Tuberculosis | 0.00044 | 0.95613 | 0.01136 | 0.03061 |
| 10 | 25 | Female | Tuberculosis | 0.01290 | 0.04836 | 0.01790 | 0.07732 |

3.1. The mRNAs of E6, LKB1, Sp1 and hTERT with qRT-PCR in bronchial brushing cells of patients with squamous cell carcinomas

| Specimen | Age | Sex | Pathologic Diagnosis | E6 | LKB1 | SP1 | hTERT |
| --- | --- | --- | --- | --- | --- | --- | --- |
| 1 | 69 | male | SCC | 0.00975 | 0.0069 | 0.04269 | 0.07802 |
| 2 | 57 | male | SCC | 0.0106 | 0.00377 | 0.05517 | 0.44751 |
| 3 | 71 | Female | SCC | 0.01663 | 0.0009 | 0.06337 | 0.63288 |
| 4 | 75 | male | SCC | 0.01398 | 0.00896 | 0.04803 | 0.16494 |
| 5 | 48 | male | SCC | 0.01428 | 0.00396 | 0.08778 | 0.25625 |
| 6 | 66 | male | SCC | 0.01871 | 0.00357 | 0.10013 | 0.4118 |
| 7 | 78 | male | SCC | 0.0116 | 0.00989 | 0.02971 | 0.18557 |
| 8 | 62 | male | SCC | 0.01563 | 0.00362 | 0.12243 | 0.70222 |
| 9 | 57 | male | SCC | 0.02352 | 0.00103 | 0.11826 | 1.53688 |
| 10 | 70 | male | SCC | 0.02342 | 0.0101 | 0.10439 | 0.07229 |
| 11 | 56 | male | SCC | 0.00446 | 0.01618 | 0.01182 | 0.03326 |
| 12 | 75 | male | SCC | 0.01031 | 0.01197 | 0.0354 | 0.00814 |
| 13 | 71 | male | SCC | 0.0164 | 0.00824 | 0.0328 | 0.57834 |
| 14 | 69 | male | SCC | 0.00975 | 0.00352 | 0.04095 | 0.94606 |
| 15 | 77 | male | SCC | 0.01845 | 0.00474 | 0.14125 | 1.33793 |
| 16 | 50 | male | SCC | 0.01136 | 0.01192 | 0.04671 | 0.1157 |
| 17 | 69 | male | SCC | 0.01286 | 0.01017 | 0.0477 | 0.20365 |
| 18 | 78 | male | SCC | 0.0261 | 0.00306 | 0.11582 | 0.92019 |
| 19 | 74 | male | SCC | 0.01224 | 0.01269 | 0.0093 | 0.06792 |
| 20 | 79 | male | SCC | 0.01499 | 0.01217 | 0.03855 | 0.12674 |

3.2. The mRNAs of E6, LKB1, Sp1 and hTERT with qRT-PCR in bronchial brushing cells of patients with squamous cell carcinomas

| Specimen | Age | Sex | Pathologic Diagnosis | E6 | LKB1 | SP1 | hTERT |
| --- | --- | --- | --- | --- | --- | --- | --- |
| 21 | 61 | male | SCC | 0.01675 | 0.00182 | 0.07536 | 0.54715 |
| 22 | 60 | male | SCC | 0.02936 | 0.00079 | 0.1432 | 1.58008 |
| 23 | 76 | male | SCC | 0.02385 | 0.00224 | 0.10789 | 0.41466 |
| 24 | 60 | Female | SCC | 0.01758 | 0.00099 | 0.10882 | 1.56917 |
| 25 | 75 | male | SCC | 0.0153 | 0.0106 | 0.05602 | 0.09025 |
| 26 | 70 | male | SCC | 0.01573 | 0.00124 | 0.06164 | 0.92659 |
| 27 | 56 | male | SCC | 0.02076 | 0.00592 | 0.13031 | 0.20166 |
| 28 | 77 | male | SCC | 0.01038 | 0.01924 | 0.04849 | 0.12851 |
| 29 | 57 | male | SCC | 0.01758 | 0.00369 | 0.09944 | 1.6245 |
| 30 | 49 | male | SCC | 0.01603 | 0.004 | 0.0733 | 0.92659 |
| 31 | 57 | male | SCC | 0.01618 | 0.00362 | 0.0819 | 1.55833 |
| 32 | 70 | male | SCC | 0.01629 | 0.0035 | 0.06561 | 0.36098 |
| 33 | 68 | male | SCC | 0.00402 | 0.0164 | 0.02756 | 0.02538 |
| 34 | 54 | male | SCC | 0.01845 | 0.00367 | 0.09278 | 0.68302 |
| 35 | 46 | male | SCC | 0.01252 | 0.01053 | 0.0359 | 0.06935 |
| 36 | 57 | male | SCC | 0.02758 | 0.0039 | 0.02969 | 0.23165 |
| 37 | 62 | male | SCC | 0.02105 | 0.00135 | 0.01351 | 1.46409 |
| 38 | 53 | male | SCC | 0.0077 | 0.01629 | 0.02592 | 0.23982 |
| 39 | 76 | male | SCC | 0.03103 | 0.00054 | 0.10882 | 0.4658 |
| 40 | 77 | male | SCC | 0.02556 | 0.00103 | 0.03615 | 4.43828 |

3.3. The mRNAs of E6, LKB1, Sp1 and hTERT with qRT-PCR in bronchial brushing cells of patients with squamous cell carcinomas

| Specimen | Age | Sex | Pathologic Diagnosis | E6 | LKB1 | SP1 | hTERT |
| --- | --- | --- | --- | --- | --- | --- | --- |
| 41 | 64 | Female | SCC | 0.02179 | 0.00099 | 0.13501 | 0.00792 |
| 42 | 58 | Female | SCC | 0.02304 | 0.00094 | 0.31425 | 0.10732 |
| 43 | 63 | male | SCC | 0.02179 | 0.00247 | 0.0364 | 1.75118 |
| 44 | 61 | male | SCC | 0.001 | 0.01314 | 0.01629 | 0.62888 |
| 45 | 60 | male | SCC | 0.00989 | 0.01252 | 0.04189 | 0.04325 |
| 46 | 56 | male | SCC | 0.02701 | 0.013064 | 0.31413 | 0.07381 |
| 47 | 50 | Female | SCC | 0.0141 | 0.01309 | 0.0135 | 0.96259 |
| 48 | 55 | male | SCC | 0.01311 | 0.00861 | 0.01438 | 0.116 |
| 49 | 70 | male | SCC | 0.02751 | 0.001 | 0.12123 | 1.53288 |
| 50 | 56 | male | SCC | 0.0103 | 0.007 | 0.0391 | 0.03326 |
| 51 | 78 | male | SCC | 0.01497 | 0.0092 | 0.064037 | 0.4028 |
| 52 | 57 | male | SCC | 0.01832 | 0.00361 | 0.10387 | 0.41389 |
| 53 | 69 | male | SCC | 0.01035 | 0.01351 | 0.0299 | 0.00799 |
| 54 | 57 | male | SCC | 0.0045 | 0.00886 | 0.02701 | 0.08002 |
| 55 | 70 | male | SCC | 0.01022 | 0.00182 | 0.0477 | 0.69332 |
| 56 | 78 | male | SCC | 0.0176 | 0.00339 | 0.035994 | 0.24842 |
| 57 | 74 | male | SCC | 0.00972 | 0.01583 | 0.04084 | 0.15994 |
| 58 | 46 | male | SCC | 0.01798 | 0.00578 | 0.07576 | 0.20185 |
| 59 | 57 | male | SCC | 0.01124 | 0.01202 | 0.04666 | 0.18398 |
| 60 | 62 | male | SCC | 0.01262 | 0.00926 | 0.02499 | 0.09935 |

3.4. The mRNAs of E6, LKB1, Sp1 and hTERT with qRT-PCR in bronchial brushing cells of patients with squamous cell carcinomas

| Specimen | Age | Sex | Pathologic Diagnosis | E6 | LKB1 | SP1 | hTERT |
| --- | --- | --- | --- | --- | --- | --- | --- |
| 61 | 53 | male | SCC | 0.0132 | 0.00789 | 0.02785 | 0.06089 |
| 62 | 71 | male | SCC | 0.01219 | 0.01259 | 0.0145 | 0.12978 |
| 63 | 74 | male | SCC | 0.015 | 0.00784 | 0.03861 | 0.09925 |
| 64 | 64 | Female | SCC | 0.01599 | 0.01309 | 0.02792 | 0.54693 |
| 65 | 68 | Female | SCC | 0.02113 | 0.00068 | 0.0989 | 1.54678 |
| 66 | 63 | male | SCC | 0.02173 | 0.00299 | 0.108782 | 0.04225 |
| 67 | 69 | male | SCC | 0.01729 | 0.00588 | 0.11534 | 0.95597 |
| 68 | 72 | male | SCC | 0.0254 | 0.00092 | 0.14278 | 0.36979 |
| 69 | 67 | male | SCC | 0.01181 | 0.00702 | 0.06151 | 0.92989 |
| 70 | 66 | male | SCC | 0.01958 | 0.0111 | 0.01112 | 0.01791 |
| 71 | 71 | Female | SCC | 0.02036 | 0.00093 | 0.08077 | 0.10692 |
| 72 | 40 | male | SCC | 0.01791 | 0.01901 | 0.0823 | 0.07489 |
| 73 | 75 | male | SCC | 0.02194 | 0.00378 | 0.0925 | 0.10045 |
| 74 | 72 | male | SCC | 0.01199 | 0.00798 | 0.0309 | 0.21941 |
| 75 | 76 | male | SCC | 0.01614 | 0.00235 | 0.0626 | 0.92026 |
| 76 | 57 | male | SCC | 0.00522 | 0.01547 | 0.0142 | 0.20111 |
| 77 | 61 | male | SCC | 0.01795 | 0.01672 | 0.09091 | 0.07493 |
| 78 | 70 | male | SCC | 0.02263 | 0.01104 | 0.08077 | 0.02538 |
| 79 | 69 | male | SCC | 0.02462 | 0.004 | 0.1408 | 0.22979 |
| 80 | 56 | male | SCC | 0.01019 | 0.00235 | 0.01348 | 0.67634 |

3.5. The mRNAs of E6, LKB1, Sp1 and hTERT with qRT-PCR in bronchial brushing cells of patients with squamous cell carcinomas

| Specimen | Age | Sex | Pathologic Diagnosis | E6 | LKB1 | SP1 | hTERT |
| --- | --- | --- | --- | --- | --- | --- | --- |
| 81 | 79 | male | SCC | 0.0081 | 0.00699 | 0.01616 | 1.3587 |
| 82 | 65 | male | SCC | 0.02498 | 0.00113 | 0.15254 | 0.57714 |
| 83 | 54 | male | SCC | 0.02162 | 0.00035 | 0.03683 | 4.33828 |
| 84 | 50 | male | SCC | 0.0211 | 0.00078 | 0.08868 | 1.6439 |
| 85 | 68 | male | SCC | 0.02278 | 0.00084 | 0.15124 | 1.47 |
| 86 | 59 | male | SCC | 0.02161 | 0.0025 | 0.11689 | 1.43 |
|  |  |  |  |  |  |  |  |
|  |  |  |  |  |  |  |  |
|  |  |  |  |  |  |  |  |
|  |  |  |  |  |  |  |  |
|  |  |  |  |  |  |  |  |
|  |  |  |  |  |  |  |  |
|  |  |  |  |  |  |  |  |
|  |  |  |  |  |  |  |  |
|  |  |  |  |  |  |  |  |
|  |  |  |  |  |  |  |  |
|  |  |  |  |  |  |  |  |
|  |  |  |  |  |  |  |  |
|  |  |  |  |  |  |  |  |
|  |  |  |  |  |  |  |  |

4. The mRNAs of E6, LKB1, Sp1 and hTERT with qRT-PCR in bronchial brushing cells of patients with adenocarcinomas

| Specimen | Age | Sex | Pathologic Diagnosis | E6 | LKB1 | SP1 | hTERT |
| --- | --- | --- | --- | --- | --- | --- | --- |
| 1 | 63 | male | AC | 0.02538 | 0.00749 | 0.19652 | 3.73213 |
| 2 | 59 | Female | AC | 0.04671 | 0.00613 | 0.18324 | 3.4822 |
| 3 | 45 | male | AC | 0.08362 | 0.00936 | 0.17237 | 2.21914 |
| 4 | 71 | male | AC | 0.0342 | 0.00704 | 0.15964 | 3.94493 |
| 5 | 63 | male | AC | 0.02486 | 0.00885 | 0.18625 | 4.02782 |
| 6 | 75 | Female | AC | 0.02486 | 0.00776 | 0.20365 | 2.62079 |
| 7 | 68 | Female | AC | 0.03258 | 0.02856 | 0.19425 | 4.85678 |
| 8 | 59 | Female | AC | 0.03082 | 0.00685 | 0.1769 | 1.70527 |
| 9 | 57 | Female | AC | 0.02778 | 0.0082 | 0.22584 | 1.94531 |
| 10 | 49 | male | AC | 0.0328 | 0.00613 | 0.19001 | 3.03143 |
| 11 | 72 | Female | AC | 0.01543 | 0.00751 | 0.19752 | 3.73434 |
| 12 | 73 | male | AC | 0.04702 | 0.0061 | 0.182 | 3.38926 |
| 13 | 68 | Female | AC | 0.09299 | 0.0094 | 0.1741 | 2.31889 |
| 14 | 57 | Female | AC | 0.0243 | 0.007 | 0.15853 | 3.93503 |
| 15 | 73 | male | AC | 0.0249 | 0.0088 | 0.18625 | 4.1367 |
| 16 | 63 | male | AC | 0.02479 | 0.008 | 0.19365 | 2.63209 |
| 17 | 59 | Female | AC | 0.0326 | 0.02752 | 0.19655 | 4.83519 |
| 18 | 71 | male | AC | 0.03281 | 0.00705 | 0.1799 | 1.72207 |
| 19 | 59 | Female | AC | 0.0374 | 0.0082 | 0.226 | 1.92131 |
| 20 | 75 | male | AC | 0.033 | 0.0061 | 0.19 | 2.94 |
