# Supplementary material for: Long-term persistent infection of HPV 16 E6 up-regulate SP1 and hTERT by inhibiting LKB1 in lung cancer cells
Source: PLoS One. 2017 Aug 16;12(8):e0182775. doi: 10.1371/journal.pone.0182775 (PMC5558957; doi:10.1371/journal.pone.0182775)
Supplement: S2 Table — (DOC) [file pone.0182775.s008.doc]

S2-Table The qRT-PCR results for E6 by Transfection and SiRNAs in H1299 and A549 cell lines

| Transfection |  |  | H1299 | SiRNAs | | A549 | |
| --- | --- | --- | --- | --- | --- | --- | --- |
| E6 | Mock | Vector | E6 | Mock |  | NS | SiE6 |
| The first time | 1.03 | 1 | 18.54 | 0.8786 |  | 1 | 0.3111 |
| The second time | 0.93 | 1 | 17.23 | 0.9013 |  | 1 | 0.2917 |
| The third time | 1.05 | 1 | 16.57 | 0.8606 |  | 1 | 0.3286 |
| LKB1 |  |  |  |  |  |  |  |
| The first time | 1.204 | 1 | 0.4946 | 1.08 |  | 1 | 1.754 |
| The second time | 0.9971 | 1 | 0.5104 | 0.96 |  | 1 | 1.407 |
| The third time | 1.0069 | 1 | 0.4807 | 1.01 |  | 1 | 1.19 |
| SP1 |  |  |  |  |  |  |  |
| The first time | 1.1794 | 1 | 3.3014 | 0.9814 |  | 1 | 0.7104 |
| The second time | 1.2105 | 1 | 3.0019 | 0.9501 |  | 1 | 0.6908 |
| The third time | 1.1699 | 1 | 3.1413 | 0.9379 |  | 1 | 0.7312 |
| hTERT |  |  |  |  |  |  |  |
| The first time | 1.1014 | 1 | 7.7714 | 0.9302 |  | 1 | 0.6701 |
| The second time | 1.0098 | 1 | 7.0609 | 0.9107 |  | 1 | 0.6434 |
| The third time | 0.9976 | 1 | 8.003 | 0.9034 |  | 1 | 0.6317 |
| SP1 activity |  |  |  |  |  |  |  |
| The first time | 0.98 | 1 | 1.51 | 1.08 |  | 1 | 0.59 |
| The second time | 0.87 | 1 | 1.32 | 1.12 |  | 1 | 0.43 |
| The third time | 1.05 | 1 | 1.26 | 1.01 |  | 1 | 0.60 |
