# Supplementary material for: Long-term persistent infection of HPV 16 E6 up-regulate SP1 and hTERT by inhibiting LKB1 in lung cancer cells
Source: PLoS One. 2017 Aug 16;12(8):e0182775. doi: 10.1371/journal.pone.0182775 (PMC5558957; doi:10.1371/journal.pone.0182775)
Supplement: S3 Table — (DOC) [file pone.0182775.s009.doc]

S3-Table The qRT-PCR results for the LKB1 by Transfection and SiRNAs in A549 and H1299 cell lines

| Transfection |  |  | A549 | SiRNAs | | H1299 | |
| --- | --- | --- | --- | --- | --- | --- | --- |
| LKB1 | Mock | Vector | LKB1 | Mock |  | NS | SiLKB1 |
| The first time | 0.97 | 1 | 16.721 | 0.854 |  | 1 | 0.243 |
| The second time | 0.934 | 1 | 15.07 | 0.881 |  | 1 | 0.280 |
| The third time | 1.06 | 1 | 14.521 | 1.040 |  | 1 | 0.300 |
| E6 |  |  |  |  |  |  |  |
| The first time | 0.9124 | 1 | 0.2781 | 1.002 |  | 1 | 2.470 |
| The second time | 0.8974 | 1 | 0.3512 | 0.815 |  | 1 | 2.135 |
| The third time | 0.9037 | 1 | 0.3214 | 0.921 |  | 1 | 2.752 |
| SP1 |  |  |  |  |  |  |  |
| The first time | 0.9431 | 1 | 0.6113 | 0.9831 |  | 1 | 2.2131 |
| The second time | 0.8973 | 1 | 0.5936 | 1.012 |  | 1 | 2.4109 |
| The third time | 0.9512 | 1 | 0.6213 | 0.9633 |  | 1 | 2.0030 |
| hTERT |  |  |  |  |  |  |  |
| The first time | 0.8712 | 1 | 0.2718 | 1.1121 |  | 1 | 1.7806 |
| The second time | 0.9011 | 1 | 0.2331 | 0.9774 |  | 1 | 1.8403 |
| The third time | 0.8413 | 1 | 0.1925 | 0.9901 |  | 1 | 1.8789 |
| SP1 activity |  |  |  |  |  |  |  |
| The first time | 0.97 | 1 | 0.55 | 0.89 |  | 1 | 1.59 |
| The second time | 0.90 | 1 | 0.71 | 1.12 |  | 1 | 1.38 |
| The third time | 0.87 | 1 | 0.63 | 1.01 |  | 1 | 1.41 |
