# Supplementary material for: Long-term persistent infection of HPV 16 E6 up-regulate SP1 and hTERT by inhibiting LKB1 in lung cancer cells
Source: PLoS One. 2017 Aug 16;12(8):e0182775. doi: 10.1371/journal.pone.0182775 (PMC5558957; doi:10.1371/journal.pone.0182775)
Supplement: S4 Table — (DOC) [file pone.0182775.s010.doc]

S4-Table The qRT-PCR results for the SP1 by SiRNAs in both A549 and H1299 cell lines

| SiRNAs |  |  | H1299 | SiRNAs | | A549 | |
| --- | --- | --- | --- | --- | --- | --- | --- |
| SP1 | Mock | NS | SiSP1 | Mock |  | NS | SiSP1 |
| The first time | 0.87 | 1 | 0.273 | 1.10 |  | 1 | 0.345 |
| The second time | 0.99 | 1 | 0.532 | 0.89 |  | 1 | 0.457 |
| The third time | 1.024 | 1 | 0.367 | 1.021 |  | 1 | 0.240 |
| hTERT |  |  |  |  |  |  |  |
| The first time | 0.9634 | 1 | 0.4543 | 0.9312 |  | 1 | 0.5943 |
| The second time | 0.9431 | 1 | 0.4716 | 0.9007 |  | 1 | 0.6003 |
| The third time | 0.9637 | 1 | 0.4903 | 0.9101 |  | 1 | 0.5742 |
